# Smaller and Denser Speech Graphs in Nondemented Patients with Progressive Supranuclear Palsy

**DOI:** 10.1155/2023/3771601

**Published:** 2023-09-25

**Authors:** Jinghong Ma, Guanyu Zhang, Xiaomin Sun, Piu Chan, Zheng Ye

**Affiliations:** ^1^Department of Neurology, Xuanwu Hospital of Capital Medical University, Beijing, China; ^2^China Institute of Sport Science, Beijing, China; ^3^Department of Neurology, Weifang People's Hospital, Weifang, China; ^4^Department of Neurobiology, Neurology and Geriatrics, Xuanwu Hospital of Capital Medical University, Beijing Institute of Geriatrics, Beijing, China; ^5^Institute of Neuroscience, Center for Excellence in Brain Science and Intelligence Technology, Chinese Academy of Sciences, Shanghai, China

## Abstract

The well-established semantic fluency test measures the ability to produce a sequence of spoken words from a particular category within a limited period of time. Like patients with Parkinson's disease (PD), patients with progressive supranuclear palsy (PSP) tend to produce fewer correct words than age-matched healthy adults. This study further examined the difference between patients with PSP and PD in their semantic fluency performance using a graph theory-based approach. Twenty-nine patients with PSP Richardson's syndrome (PSP-RS), thirty-eight patients with PD, and fifty-one healthy controls (HC) were recruited. All participants completed a standard semantic fluency test (animals). Their verbal responses were recorded, transcripted, and transformed into directed speech graphs. The speech graphs of the PSP-RS group showed higher density, shorter diameter, and shorter average shortest path than those of the PD and HC groups. It indicates that the PSP-RS group produced smaller and denser speech graphs than the PD and HC groups. In the PSP-RS group, moreover, the average shortest paths of the speech graphs correlated with the severity of motor symptoms. This study shows the potential of the graph theory-based approach in distinguishing the semantic fluency performance of nondemented patients with PSP-RS and PD.

## 1. Introduction

The well-established semantic fluency test measures the ability to produce a sequence of spoken words from a particular category within a limited period of time. Semantic disfluency is a common problem in progressive supranuclear palsy (PSP) and Parkinson's disease (PD). Patients with PSP or PD tended to generate fewer correct words than age-matched healthy adults in the semantic fluency test [1, 2].

PSP is defined by abnormal intracerebral tau-protein aggregations with motor, ocular, and cognitive symptoms. An epidemiological study from the United Kingdom showed that PSP is a rare disease with a prevalence rate of 5-7/100,000 [3]. The most common clinical phenotype of PSP is Richardson's syndrome (PSP-RS), characterized as the combination of early-onset postural instability and falls with vertical ocular motor dysfunction [4]. The PSP and PD are complex neurodegenerative disorders characterized by motor and non-motor symptoms, so it may be difficult to distinguish PSP from PD, especially at the early stages [5]. Therefore, early detection is crucial to identify patients for appropriate clinical interventions and support.

Clustering and switching analyses were used to quantify responses in verbal fluency tests [6]. Clustering analysis segmented correct words into a certain cluster (subcategory) according to the semantic relatedness between words. Switching is the ability to shift efficiently between clusters. For example, a participant may begin with vertebrate animals (e.g., tiger and lion) and then switch to invertebrate animals (e.g., frog, snake). This approach had two primary parameters: the mean cluster size, which is the average number of words from the same subcategory (e.g., vertebrate animals), and the number of switches between clusters. The PSP and PD patients produced smaller clusters and switched less than healthy adults in verbal fluency tasks [7, 8].

The clustering and switching analyses relied heavily on the experimenters' subjective judgment of cluster segmentation. An automated computational approach was proposed by Farzanfar et al. [9], in which each correct word is represented as a vector according to the semantic corpora and the semantic relatedness between paired words is represented as the cosine of the angle between the corresponding vectors. The words with higher semantic relatedness values than the predetermined threshold were segmented into the same cluster, and those with lower values were segmented into different clusters. However, the automated computational approach was inconsistent with the experimenter-dependent method in calculating clusters and was much influenced by the predetermined threshold.

An objective method is derived from graph theory. Graph theory has been used to analyze the topological changes of brain networks in various neurodegenerative disorders [10, 11]. Bertola et al. [12] used graph theory to analyze semantic fluency data and found that the speech graphs of patients with Alzheimer's disease (AD) had a higher density, shorter diameter, and shorter average shortest path than those of patients with mild cognitive impairment, illustrating that the speech graphs become smaller and denser with the decline of general cognition. Our recent study also confirmed that the speech graphs of PD patients were smaller and denser than those of healthy controls but larger and sparser than those of AD patients [13]. As a sensitive approach, we hypothesize that the graph analysis can extract more topological features, which potentially contributes to tell the difference between PSP and PD patients without dementia.

**Figure 1:**
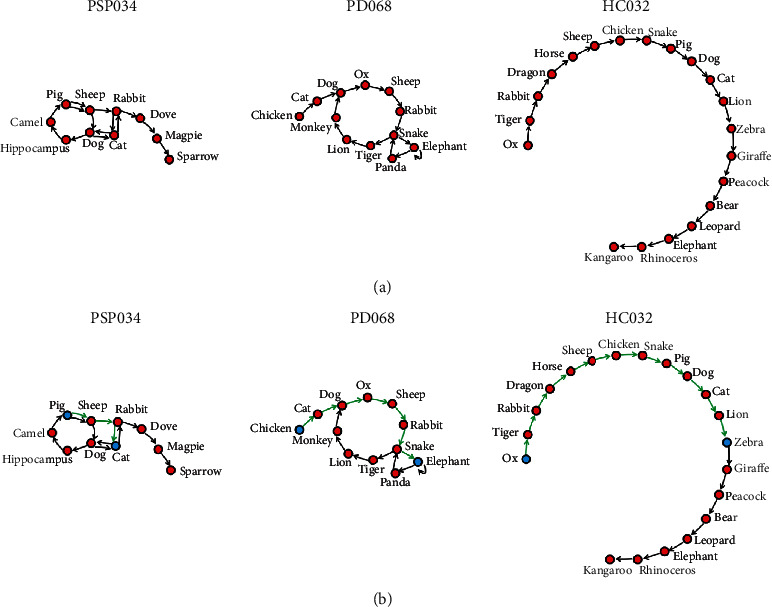
(a) Directed speech graphs of three representative participants. PSP034, a patient with progressive supranuclear palsy-Richardson's syndrome; PD068, a patient with Parkinson's disease; HC032, a healthy control subject. (b) The shortest path (green) between two nodes (blue) in the three participants.

In this study, we examined the difference between patients with PSP-RS and PD in their semantic fluency performance using a graph theory-based approach. All participants completed a standard semantic fluency test (animals). We transformed participants' verbal responses into directed speech graphs. Each correct word was represented as a node, and the connection between consecutive words was represented as an arc (Figure 1). First, we detected group differences in standard (the number of correct words, repetitions, incorrect words, metalinguistic reference, and metacognitive reference) and graph parameters (global characteristics of speech graphs, including density, diameter, and average shortest path). Second, in PSP-RS, we explored whether the standard or graph parameters correlated with the severity of non-motor or motor symptoms.

## 2. Materials and Methods

This study was approved by the ethics committee of the Xuanwu Hospital according to the Declaration of Helsinki. Each participant signed a written informed consent before participating in this study.

### 2.1. Patients and Clinical Assessments

We included 29 patients with probable PSP-RS (Movement Disorder Society Clinical Diagnostic Criteria for Progressive Supranuclear Palsy [14]) at the Xuanwu Hospital between 2022 and 2023. Inclusion criteria were (1) Hoehn and Yahr stages 1 to 3, (2) age 40 to 75 years, (3) education ≥ 6 years, and (4) Mandarin Chinese speaking. Exclusion criteria were (1) a history of epilepsy, stroke, or brain injury; (2) alcohol or drug abuse; (3) possible current depression (Beck Depression Inventory-II, BDI − II > 7) or intake of antidepressants; and (4) possible dementia (Montreal Cognitive Assessment, MoCA < 21/30) or intake of antidementia drugs.

All patients were parkinsonian and did not respond to levodopa. They were assessed on their regular antiparkinsonian drugs, including levodopa (*N* = 23), selegiline (*N* = 7), amantadine (*N* = 4), pramipexole (*N* = 4), rasagiline (*N* = 4), and piribedil (*N* = 3). The levodopa equivalent daily dose was calculated using the equation of Tomlinson et al. [15]. The severity of motor and non-motor symptoms was evaluated with the Movement Disorder Society-sponsored revision of the Unified Parkinson's Disease Rating Scale (MDS-UPDRS) Part III subscale and the Non-motor Symptoms Scale (NMSS), respectively.  Table 1 shows demographic and clinical features and neuropsychological measures. Although there was a significant difference among the three groups in MoCA, there was no difference between the PSP-RS and PD groups (*p* = 0.155).

### 2.2. Two Control Groups

We included two control groups: 38 age- and education-matched patients with idiopathic PD (Movement Disorder Society Clinical Diagnostic Criteria for Parkinson's Disease [16]) from Xuanwu Hospital and 51 age- and education-matched healthy controls (HC) from local communities.

As a positive control group [13], the inclusion and exclusion criteria of PD were the same as PSP-RS. All patients were assessed on their regular antiparkinsonian drugs, including levodopa (*N* = 21), pramipexole (*N* = 16), selegiline (*N* = 15), amantadine (*N* = 6), piribedil (*N* = 6), entacapone (*N* = 2), and rasagiline (*N* = 1). They completed the same clinical and neuropsychological assessments as PSP-RS patients.

For the HC group, exclusion criteria were (1) a history of significant neurological or psychiatric disorders, (2) alcohol or drug abuse, (3) possible current depression, and (4) possible dementia or mild cognitive impairment (MoCA < 26/30). They completed the same assessments for cognition, mood, and sleep as patients.

### 2.3. Standard and Graph Analyses

All participants completed a standard semantic fluency test (animals). We recorded and transcripted their verbal responses.

For the standard analysis, we defined five parameters: (1) the number of correct words; (2) the number of repetitions; (3) the number of incorrect words (e.g., leaf); (4) metalinguistic reference: the number of times participants talked about their responses (e.g., “Is pig the correct answer?”); and (5) metacognitive reference: the number of times participants talked about their memory and asked time remaining (e.g., “I really can't think of any.”).

For the graph analysis, we transformed participants' verbal responses into directed speech graphs with SpeechGraph software [12, 13], in which each correct word was represented as a node and each temporal link between sequential words was represented as an arc (Figure 1(a)). We computed three typical graph parameters, including the density, diameter, and average shortest path. The density is the number of arcs divided by the maximum possible number of arcs. The diameter is the length of the longest shortest path between two nodes (Figure 1(b)). The average shortest path is the average length of the shortest path between two nodes, also known as the characteristic measure of the graph.

More details were described in our recent study [13].

### 2.4. Statistical Analysis

Data were analyzed with IBM SPSS Statistics 20. First, we examined group differences in the standard and graph parameters using one-way ANOVAs (two-tailed, *p* < 0.006, Bonferroni's correction for eight tests). The ANOVA had a factor group (HC, PD, and PSP-RS) and covariates age and education. Significant group differences were followed by two-sample *t*-tests.

Second, in PSP-RS, we examined whether the severity of motor or non-motor symptoms (MDS-UPDRS Part III subscore or NMSS score) correlated with the standard and graph parameters that showed group differences using linear stepwise regression models (two-tailed, *p* < 0.025, Bonferroni's correction for two models).

## 3. Results

### 3.1. Group Differences in Standard Parameters

**Figure 2:**
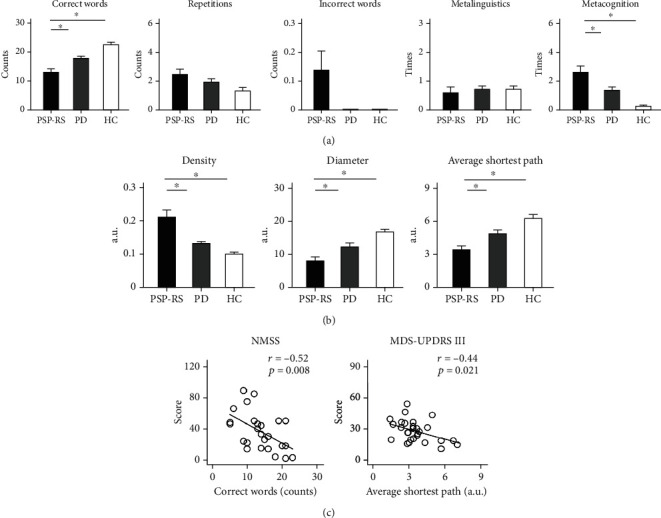
(a) Means and standard errors of correct words, repetitions, incorrect words, metalinguistic reference, and metacognitive reference in patients with progressive supranuclear palsy-Richardson's syndrome (PSP-RS), patients with Parkinson's disease (PD), and healthy controls (HC). Asterisks, *p* < 0.05. (b) Means and standard errors of graph density, diameter, and average shortest path in each group. Asterisks, *p* < 0.05. (c) In PSP-RS, the number of correct words was correlated with the severity of nonmotor symptoms (NMSS score). The average shortest paths of speech graphs correlated with the severity of motor symptoms (MDS-UPDRS III score).

Figure 2(a) shows the standard parameters in each group. Group differences were found in the number of correct words (*F*(2, 113) = 24.31, *p* < 0.001, *η*_*p*_^2^ = 0.30), number of incorrect words (*F*(2, 113) = 6.22, *p* = 0.003, *η*_*p*_^2^ = 0.10), and metacognitive reference (*F*(2, 113) = 27.32, *p* < 0.001, *η*_*p*_^2^ = 0.33), but not in the number of repetitions (*F*(2, 113) = 2.67, *p* = 0.074, *η*_*p*_^2^ = 0.05) and metalinguistic reference (*F* < 1). The PSP-RS group generated fewer correct words than the PD (*t*(65) = −3.80, *p* < 0.001) and HC groups (*t*(78) = −7.04, *p* < 0.001). The PSP-RS group talked more about their memory and time remaining than the PD (*t*(65) = 2.63, *p* = 0.012) and HC groups (*t*(78) = 5.46, *p* < 0.001). Only the PSP-RS group generated incorrect words (*N* = 4).

### 3.2. Group Differences in Graph Parameters

Figure 2(b) shows the graph parameters in each group. Group differences were found in the density (*F*(2, 113) = 19.54, *p* < 0.001, *η*_*p*_^2^ = 0.26), diameter (*F*(2, 113) = 16.72, *p* < 0.001, *η*_*p*_^2^ = 0.23), and average shortest path (*F*(2, 113) = 18.08, *p* < 0.001, *η*_*p*_^2^ = 0.24). The speech graphs of the PSP-RS group showed higher density (PD: *t*(65) = 3.31, *p* = 0.002; HC: *t*(78) = 4.70, *p* < 0.001), shorter diameter (PD: *t*(65) = −3.40, *p* = 0.001; HC: *t*(78) = −6.93, *p* < 0.001), and shorter average shortest path than those of the PD and HC groups (PD: *t*(65) = −3.51, *p* = 0.001; HC: *t*(78) = −7.18, *p* < 0.001). In other words, the speech graphs of the PSP-RS group were smaller and denser than those of the PD and HC groups.

### 3.3. Correlations between the Severity of Non-motor and Motor Symptoms and Standard and Graph Parameters in PSP-RS

Figure 2(c) shows the correlations between the severity of non-motor and motor symptoms and standard and graph parameters in PSP-RS. The stepwise regression model for the NMSS score (*F*(1, 23) = 8.49, *p* = 0.008, *R*^2^ = 0.27) included the number of correct words (beta = −2.45, *t* = −2.91, *p* = 0.008) but removed the number of incorrect words (|*t*| < 1), metacognitive reference (|*t*| < 1), density (|*t*| < 1), diameter (|*t*| < 1), and average shortest path (|*t*| < 1). PSP-RS patients with more severe non-motor symptoms tended to generate fewer correct words.

The stepwise regression model for the MDS-UPDRS Part III subscore (*F*(1, 26) = 6.08, *p* = 0.021, *R*^2^ = 0.19) included the average shortest path (beta = −3.28, *t* = −2.47, *p* = 0.021) but removed the number of correct words (|*t*| < 1), number of incorrect words (|*t*| < 1), metacognitive reference (|*t*| < 1), density (|*t*| < 1), and diameter (|*t*| < 1). PSP-RS patients with more severe motor symptoms tended to produce smaller and denser speech graphs.

## 4. Discussion

In this study, we revisited the semantic disfluency in nondemented patients with PSP-RS. We replicated previous findings that PSP patients generated fewer correct words than the PD patients and healthy controls [17, 18]. We examined the topology of participants' speech graphs using a graph theory-based approach and found that PSP-RS patients produced smaller and denser speech graphs than the PD patients and healthy controls. To be specific, the speech graphs of PSP-RS patients showed higher density, shorter diameter, and shorter average shortest path than those of the PD patients and healthy controls. In the PSP-RS group, moreover, the numbers of correct words and average shortest paths of speech graphs correlated with the severity of non-motor and motor symptoms, respectively. The PSP-RS patients who generated fewer correct words exhibited more severe non-motor symptoms in daily living, and those who produced smaller and denser speech graphs exhibited more severe motor symptoms.

The graph analysis revealed new features of semantic disfluency and showed good discrimination between PSP and PD. What is more, our study suggested that graph analysis was more sensitive than standard analysis. For example, the standard analysis showed that there was no difference between patients with PSP-RS and healthy controls in repetitions, but the graph analysis indicated that the PSP-RS patients produced more repetitive words than healthy controls, as shown by denser and smaller speech graphs. The topological change of speech graphs in patients with PSP-RS suggested that we should focus on repetitions in semantic fluency. The repetition might reflect the deficits in word selection and speech programming in semantic fluency.

A selection mechanism will be applied to produce verbal responses that meet the instruction and inhibit the retrieval of inappropriate words. Previous studies have shown that the left inferior frontal gyrus (LIFG) plays a modulatory role in this process. Thompson-Schill et al. proposed that the LIFG is critical for selecting relevant options in the face of competing alternatives [19]. Hirshorn and Thompson-Schill also confirmed that increased selection demands are associated with greater activation in the LIFG in semantic fluency tasks [20]. It has been suggested that the basal ganglia participate in programming and initiation processes. Watson and Montgomery used microelectrodes to record subthalamic neuronal activity in humans and observed that the activation was greater in speech programming but was lower in speech production [21]. Tröster et al. showed worse performance of verbal fluency approximately 4 months after unilateral pallidotomy in patients with PD, suggesting that frontal-basal ganglionic circuits were involved in word retrieval processes [22].

Compared to PD patients, the profound speech impairment in PSP patients may be due to more severe frontal-basal ganglionic pathology [23]. A functional magnetic resonance imaging force production paradigm analysis showed that in PSP, the frontal regions are underactive and functional activity of the basal ganglia and cortical motor areas is weakened as compared with PD [24]. Another study used semiquantitative analysis of neuronal loss and reported that the deficits of speech production were correlated with the degree of neuronal loss in the substantia nigra in patients with PSP [25].

The relationship between the semantic disfluency and the severity of motor or non-motor symptoms in PSP has been confirmed in the previous studies. The initiation/perseveration function of the semantic fluency task could predict gait velocity in PSP [26]. In addition, PD patients with advanced stages of disease (Hoehn and Yahr Scale), right hemibody onset of motor symptoms, or severe sleep disorders scored lower in semantic fluency tasks [27, 28]. Our results were consistent with the previous studies. The severity of nonmotor and motor symptoms was correlated with standard and graph parameters in PSP-RS, respectively, so that doctors could prejudge the symptom severity of PSP patients through their performances on the semantic fluency task.

This study has limitations. First, many studies reported that neuropsychological tests could differentiate the PSP phenotypes [29, 30]. Given the small sample size of PSP patients with other phenotypes, we only include patients with PSP-RS to increase homogeneity so that this study could not examine the difference between PSP-RS and other phenotypes. Second, although results showed that graph analysis of semantic fluency test could discriminate between PSP-RS and PD patients at the early stages, it is more valuable for verbal fluency tests to correctly discriminate early grey cases with the aid of SpeechGraph software. Future follow-up studies could examine whether graph analysis of verbal fluency tests can correctly discriminate early grey cases. Third, previous pharmacological studies confirmed the beneficial effect of monoamine oxidase type-B inhibitor on verbal fluency in nondemented patients with PD [31]. Our study cannot achieve this goal due to the small sample size. Future pharmacological studies can explore the effect of antiparkinsonian drugs on PSP patients' speech graphs.

## 5. Conclusion

In this study, we used graph theory to analyze the topological change of speech graphs by the semantic fluency test in patients with PSP-RS. The speech graphs of PSP-RS patients were smaller and denser than those of the PD patients and healthy controls, indicating the potential of the graph theory-based approach in distinguishing the semantic fluency performance of nondemented patients with PSP-RS and PD. Moreover, PSP-RS patients who generated fewer correct words exhibited more severe nonmotor symptoms, and those who produced smaller and denser speech graphs exhibited more severe motor symptoms.

## Figures and Tables

**Table 1:** Demographic and clinical features and neuropsychological measures of patients and healthy controls (means, standard deviations, and group differences).

Features/measures	PSP-RS (*N* = 29)	PD (*N* = 38)	Healthy controls (*N* = 51)	Group differences (*p* values)
Male : female	16 : 13	17 : 21	26 : 25	0.845
Age (years)	61.9 (6.2)	60.4 (7.1)	60.4 (6.9)	0.607
Education (years)	10.7 (3.0)	11.3 (2.9)	11.8 (2.1)	0.197
Motor symptoms				
MDS-UPDRS III: motor examination	27.5 (10.6)	23.4 (12.2)	—	0.162
Hoehn and Yahr Scale	2.4 (0.6)	2.1 (0.8)	—	0.125
Disease duration (years)	1.4 (2.1)	2.0 (2.0)	—	0.202
Duration of motor symptoms (years)	2.7 (2.1)	3.5 (2.5)	—	0.179
Levodopa equivalent daily dose (mg/day)	332.8 (259.2)	320.6 (275.1)	—	0.855
Non-motor functions				
Non-motor Symptoms Scale	36.5 (24.5)	26.6 (21.6)	—	0.172
Beck Depression Inventory-II	3.4 (2.0)	3.0 (2.0)	2.4 (1.8)	0.074
REM Sleep Behaviour Disorder Screening Questionnaire	1.8 (1.0)^a^	3.8 (1.4)^b^	2.1 (2.1)	0.001^∗^
Epworth Sleep Scale	4.7 (5.1)	4.2 (3.9)	3.1 (2.0)	0.165
Montreal Cognitive Assessment	23.5 (2.1)^b^	24.3 (2.6)^b^	27.9 (1.4)	<0.001^∗^

Note: PSP-RS: progressive supranuclear palsy-Richardson's syndrome; PD: Parkinson's disease; MDS-UPDRS: Movement Disorder Society-sponsored revision of the Unified Parkinson's Disease Rating Scale. Group differences, *p* values of one-way ANOVAs or the Kruskal-Wallis one-way ANOVAs as appropriate. Asterisks (^∗^), a significant difference (two-tailed, *p* < 0.004, Bonferroni's correction for thirteen tests). Post hoc two-sample *t*-tests, *p* < 0.004. ^a^Compared with patients with PD. ^b^Compared with healthy controls.

## Data Availability

Data have been uploaded to the Figshare database (https://figshare.com/articles/dataset/PD_HO_v2_xls/13607441).
